# The ENCOMPASS framework: a practical guide for the evaluation of public health programmes in complex adaptive systems

**DOI:** 10.1186/s12966-022-01267-3

**Published:** 2022-03-28

**Authors:** Angie Luna Pinzon, Karien Stronks, Coosje Dijkstra, Carry Renders, Teatske Altenburg, Karen den Hertog, Stef P. J. Kremers, Mai J. M. Chinapaw, Arnoud P. Verhoeff, Wilma Waterlander

**Affiliations:** 1 Department of Public and Occupational Health, Amsterdam Public Health Research Institute, Amsterdam UMC, University of Amsterdam, Meibergdreef 9, 1105AZ Amsterdam, The Netherlands; 2 Department of Health Sciences, Amsterdam Public Health Research Institute, Vrije Universiteit Amsterdam, 1081HV Amsterdam, The Netherlands; 3 Department of Public and Occupational Health, Amsterdam Public Health Research Institute, Amsterdam UMC, Vrije Universiteit Amsterdam, Van der Boechorststraat 7, 1081BT Amsterdam, The Netherlands; 4 Amsterdam Healthy Weight Approach, Public Health Service (GGD), 1018WT Amsterdam, The Netherlands; 5 Department of Health Promotion, NUTRIM School of Nutrition and Translational Research in Metabolism, Maastricht University, 6229ER Maastricht, The Netherlands; 6 Sarphati Amsterdam, Public Health Service (GGD), 1018WT Amsterdam, The Netherlands; 7 Department of Sociology, University of Amsterdam, 1018WV Amsterdam, The Netherlands

**Keywords:** Overweight and obesity, Whole-of-systems approaches, Systems thinking, Complex systems, Public health, Evaluation, Practice, Participatory action research

## Abstract

**Background:**

Systems thinking embraces the complexity of public health problems, including childhood overweight and obesity. It aids in understanding how factors are interrelated, and it can be targeted to produce favourable changes in a system. There is a growing call for systems approaches in public health research, yet limited practical guidance is available on how to evaluate public health programmes within complex adaptive systems. The aim of this paper is to present an evaluation framework that supports researchers in designing systems evaluations in a comprehensive and practical way.

**Methods:**

We searched the literature for existing public health systems evaluation studies. Key characteristics on how to conduct a systems evaluation were extracted and compared across studies. Next, we overlaid the identified characteristics to the context of the Lifestyle Innovations Based on Youth Knowledge and Experience (LIKE) programme evaluation and analyzed which characteristics were essential to carry out the LIKE evaluation. This resulted in the Evaluation of Programmes in Complex Adaptive Systems (ENCOMPASS) framework.

**Results:**

The ENCOMPASS framework includes five iterative stages: (1) adopting a system dynamics perspective on the overall evaluation design; (2) defining the system boundaries; (3) understanding the pre-existing system to inform system changes; (4) monitoring dynamic programme output at different system levels; and (5) measuring programme outcome and impact in terms of system changes.

**Conclusions:**

The value of ENCOMPASS lies in the integration of key characteristics from existing systems evaluation studies, as well as in its practical, applied focus. It can be employed in evaluating public health programmes in complex adaptive systems. Furthermore, ENCOMPASS provides guidance for the entire evaluation process, all the way from understanding the system to developing actions to change it and to measuring system changes. By the nature of systems thinking, the ENCOMPASS framework will likely evolve further over time, as the field expands with more completed studies.

## Background

Public health problems such as childhood overweight and obesity, tobacco use and type 2 diabetes can be considered complex problems, given the multiple and dynamic factors that drive them. Those factors range from individual behaviours (such as levels of physical activity) to more upstream determinants (such as urban design). The factors are interconnected [1, 2]. Because of this complexity, the application of systems thinking is of growing interest in public health, both for gaining an understanding of public health problems from a complexity perspective – looking at the ‘bigger picture’ – and for developing programmes that operate within complex adaptive systems and thereby take into account the inherent complexity of the real world [3–6].

A complex adaptive system can be defined using various terms. Table 1 explains the terms used in this paper. At its most basic level, a *system* is composed of multiple parts (including actors, organisations or processes) that are interconnected [11, 12]. A *complex adaptive system* is emergent; it is more than the sum of its parts [7]. Examples of complex adaptive systems include communities, schools and the ecosystem. Complex adaptive systems possess different properties. A complex adaptive system is dynamic, which means that its parts and interconnections operate in such a way that they produce their own pattern of behaviour over time. This system behaviour is determined by a system’s *purpose* [5, 12]. For example, secondary schools could be seen as complex adaptive systems whose main purpose is to provide education to adolescents.

**Table 1:** Glossary of system definitions^a^

Concept	Definition
Adaptation	Adjustments in the behaviour of a system or programme in response to new conditions.
Boundary (system boundary)	System boundaries define what components of a system need to be included, or can be excluded, to understand the system under study.
Causal loop diagram (CLD)	Visual representation of a system consisting of closed loops of causal influences that capture how components of a system interrelate.
Complex adaptive system	A system is a set of individual components that are interconnected. A complex adaptive system is more than the sum of its parts: the system as a whole has different properties to those that can be found in individual components of the system.
Disruption	A significant event that prevents a system from continuing its normal trajectory or behaviour.
Dynamic	A complex adaptive system is dynamic – the behaviour of the system changes over time.
Emergence	Properties of a complex system that cannot be directly predicted from the elements within it and are more than just the sum of its parts.
Feedback loop	Feedback occurs when the output of a causal influence also serves as an input of that causal influence. A feedback loop is a sequence of components and interconnections that creates a closed loop of causal influences.
Group model building (GMB)	Methodology for developing models in which people as a group participate actively and simultaneously in building a causal loop diagram.
Leverage points	Places in a system where a small change could lead to a large change in the system’s behaviour.
Non-linear relationship	Relationship between two components in a system, in which a change in the first component (‘independent variable’) does not produce a proportional effect in the second component (‘dependent variable’).
Self-organisation	The ability of a complex adaptive system to structure itself, to create a new structure, to learn, or to diversify by local interactions between individual components, rather than by external forces.
Social network analysis (SNA)	Technique used to describe and analyse patterns of social interaction between different entities (e.g. people, organisations).
System behaviour	Individual components in complex adaptive systems are interconnected in such a way that they together produce a distinct pattern of behaviour over time. The system’s function or purpose is what ultimately determines how the system as a whole will behave.
System dynamics	In system dynamics, models (e.g. causal loop diagrams) are built that represent the dynamic complexity of high-level phenomena.
Systems thinking	A way of conceptualising and making sense of the world through the application of systems concepts such as adaptation, feedback loops and emergence.
Uncertainty	Under conditions of complexity, processes and outcomes cannot be predicted, be controlled or be known in advance.
Unintended consequences	Unplanned (and typically undesirable) side-effects of actions in a system, often occurring after a time delay.

^*a*^*Definitions were extracted from Meadows & Wright* [7]*, Patton* [8]*, McGill* et al. [5]*, Garcia* et al. [9] *and Ford* [10]

Complex adaptive systems are open to influences from within the system and from its wider environment. They *adapt* their behaviour in response to those influences. For instance, a newly introduced national policy that makes physical education compulsory throughout secondary school, with the aim of encouraging adolescents to be more physically active, could lead to many logistical challenges such as hiring new staff, building sport facilities and readjusting class schedules. The interactions of parts within complex adaptive systems are *non-linear*, meaning that changes in one part of the system may lead to small or large effects (expected or unexpected) on other parts of the system, or on the system as a whole [8, 13, 14]. Adolescents that oppose physical education being compulsory may, for instance, embark on unhealthy behaviours like smoking on school premises as a way of rebelling (hence, an unexpected effect of the newly introduced national policy). Non-linearity also means that differences between systems may lead to different outcomes over time [12]. While secondary schools in a certain community may look similar, the quality of physical education they provide to the same community may differ. Lastly, complex adaptive systems cannot be controlled, predicted or fully known (*uncertainty*) [5].

Although the listed properties of complex adaptive systems are increasingly being acknowledged when applying a systems perspective in public health programmes, there is still little guidance available as to what such a systems approach will entail for the *evaluation* of programmes that are implemented in the system according to these principles. For example, a particular programme may advocate that the purpose of secondary schools should be not only to educate adolescents (the purpose of the *current* system) but also to contribute to ‘raising’ healthy adolescents (the *new* system’s purpose). Such a programme would need to work on *transforming* the prevailing system’s purpose, so that raising healthy adolescents will no longer be the mere responsibility of parents, but of the community as well. To achieve this aim, such a programme cannot be designed just to deliver a specific package of activities at a single level (such as a healthy school programme), but it should target the different levels of the system, following the principles of system dynamics outlined here.

From this illustration it is evident that the evaluation of programmes following the principles of system dynamics would present some challenges, for instance in terms of the applicable methods or the kinds of evidence such evaluation should yield and how these should be interpreted [5]. A 2019 review of the effectiveness of programmes in complex adaptive systems by Bagnall and colleagues concluded that, although systems approaches were increasingly being applied in public health, most of the reviewed studies did not include a systems evaluation strategy for their approaches, and also that substantial heterogeneity existed between studies in terms of research design and outcomes [15]. In particular, few programmes had been explicitly designed with an a priori recognition of the problem as the outcome of a system and rarely approached design or implementation of the programme activities from a systems perspective. Consequently, the review found little recognition of properties inherent in a complex adaptive system (such as non-linear relationships, feedback loops, and dynamic properties) and there was little attention to the evaluation and reporting of system outcomes [15].

A systems evaluation should include an adaptive approach [16] which is able to continuously capture changes in the system, which is receptive to new information that emerges from a better understanding of the system, and which allows for inclusion of feedback and of unexpected outcomes. Evidently, this adaptive approach does not very well suit conventional study designs, such as randomized controlled trials, because there is neither a pre-specified programme, nor a ‘control system’, nor pre-specified outcomes.

Only limited guidance exists on how the principles of system dynamics can or should be included in the evaluation of public health programmes in complex adaptive systems [5, 9, 17, 18]. Two examples include a recent methodological review by McGill et al., on the evaluation of public health interventions from a complex systems perspective [5] and the framework for developing and evaluating complex interventions from the Medical Research Council [18] (for more details we refer to Table 2). These guidelines tend to however remain quite theoretical, provide insufficient guidance on how to conduct a systems evaluation in practice, or discuss only certain parts of the evaluation process.

**Table 2:** Key characteristics of systems evaluation studies that inform the ENCOMPASS framework

Title	Evaluating system change: a planning guide – Hargreaves 2010 [19].	Applying complexity theory: A review to inform evaluation design – Walton 2014 [12].	Guidance on systems approaches to local public health evaluation – Part 2: What to consider when planning a systems evaluation – Egan et al. 2019 [17].	Evaluation of public health interventions from a complex systems perspective: A research methods review – McGill et al. 2021 [5].	An action-oriented framework for systems-based solutions aimed at childhood obesity prevention in US Latinx and Latin American populations – Garcia et al. 2021 [9].	A new framework for developing and evaluating complex interventions: Update of Medical Research Council guidance – Skivington et al. 2021 [18].
**Study aim**	To provide guidance on how to plan the evaluation of a system change intervention	To identify themes to take into account when applying a complexity lens to evaluation	To provide guidance on evaluating public health interventions that take a systems approach	To classify the various types of methods employed in systems evaluations and examining the evidence produced by those methods	To provide a roadmap for designing, implementing, evaluating and sustaining whole-of-community systems changes interventions	To support researchers in identifying key intervention questions and in designing and conducting research from a systems perspective
**Identified theoretical elements**		(1) Understanding the system (2) Considering emergence and other complexity concepts (3) Defining level and unit of analysis (4) Appropriately timing the evaluation (5) Considering participatory methods (6) Using multiple and mixed methods (7) Defining theories of change	(1) Defining evaluation questions and focus (2) Understanding the system (3) Identifying levers of change			(1) Considering context (2) Developing, refining, testing and retesting programme theory (3) Engaging stakeholders (4) Identifying key uncertainties (5) Refining intervention
**Identified evaluation stages**	(1) Understanding the conditions and dynamics of the system: boundaries, relationships and multiple perspectives (2) Understanding the elements and dynamics of the intervention: theory of change, intended outcomes, and the monitoring of unintended outcomes (3) Determining the users, purposes and methods of the evaluation			(1) Theorising (2) Prediction (simulation) (3) Process evaluation (4) Impact evaluation (5) Further prediction (simulation)	(1) Fostering multisectoral team (2) Mapping the system, context and drivers (3) Envisaging system-wide changes (4) Effecting system-wide changes (5) Monitoring, learning and adapting (6) Scaling and sustaining	(1) Developing intervention or identifying intervention (2) Feasibility and pilot testing (3) Evaluation (4) Implementation
**Examples of methods**	- Agent-based modelling - Appreciative inquiry, reflective practice - Case studies, interviews, focus groups - Document reviews - Observations - Outcome mapping, concept mapping - System pattern analyses - Time-trend analysis - Tracking activities	- Agent-based modelling - Interviews - Network analysis - Qualitative comparative analysis - Time-trend analysis	- Agent-based modelling - Concept mapping - Network analysis - Qualitative research with a systems lens - Systems dynamic modelling	- Network analysis - System framing - System mapping - System modelling	- Adaptive policy approaches - Causal loop diagram - Group model building - Interviews - Network analysis - Scenario modelling	- Group model building - Social network analysis - System modelling

In the present study, we aim to integrate key characteristics of existing systems evaluation guidelines and apply these characteristics to the context of the Lifestyle Innovations Based on Youth Knowledge and Experience (LIKE) programme. This will enable us to arrive at a framework that can support researchers in designing public health systems evaluations in comprehensive and practical ways.

## Methods

The Evaluation of Programmes in Complex Adaptive Systems (ENCOMPASS) framework was developed in a three-step process described below. While these steps are described as a linear course of action, the process was rather iterative, in particular Step 3 (see details below).

### Step 1. Literature search of systems evaluation studies

The first step involved a pragmatic literature search of existing studies that provided guidance on how to design a systems evaluation. For a study to be included, the following criteria had to be met: (1) the study acknowledged complexity and hence discussed programmes in complex adaptive systems; (2) the study provided guidance on how to design or conduct a systems evaluation; and (3) the study included examples of public health problems. Studies that met those criteria were identified through an electronic search (in PubMed and Google Scholar). Search terms included: systems thinking or system dynamics or complexity theory or complex system; and evaluation or evaluation theory or systems evaluation; and public health. In addition, authors of the present study suggested a number of articles for potential inclusion. A total of six studies were ultimately included (Table 2).

### Step 2. Extracting key characteristics of included studies

The second step involved making an overview of the key characteristics discussed by the six included studies pertaining to how a systems evaluation should be carried out (Table 2). In those studies we could broadly distinguish two approaches with regard to the type of guidance provided for conducting a systems evaluation. Two studies discussed specific *theoretical elements* that a systems evaluation should include [12, 17]; three studies identified different *stages* of a systems evaluation [5, 9, 19]; and one study identified both theoretical elements and evaluation stages [18]. All six suggested various methods, including system dynamics methods, that may be included in a systems evaluation. Worthy of note is that the use of *single* system dynamics methods does not take full account of the complex, emergent and unpredictable nature of systems [5]. That would require application of *multiple methods* that enable a more complete understanding of the system as a whole [20]. Such an understanding is necessary to develop and evaluate programmes that aim to transform a particular complex adaptive system.

### Step 3. Development of the ENCOMPASS framework

Table 2 provides an overview of the key characteristics: (a) theoretical elements; (b) evaluation stages; and (c) methods that a systems evaluation should include, based on the six analysed studies. It is evident that there is some overlap between the studies, but also that none of their frameworks covers all stages of a systems evaluation that would range from gaining an understanding of the pre-existing system to measuring how a system changes after implementation of a particular programme or activities. In addition, the existing frameworks provide little practical guidance on how a systems evaluation can be conducted from start to finish. In our third and last step, our aim was to therefore integrate the theoretical elements and stages of the six different studies into a single overarching framework. We did so by applying the theoretical elements and evaluation stages to the context of the Lifestyle Innovations Based on Youth Knowledge and Experience (LIKE) programme.

**Fig. 1:**
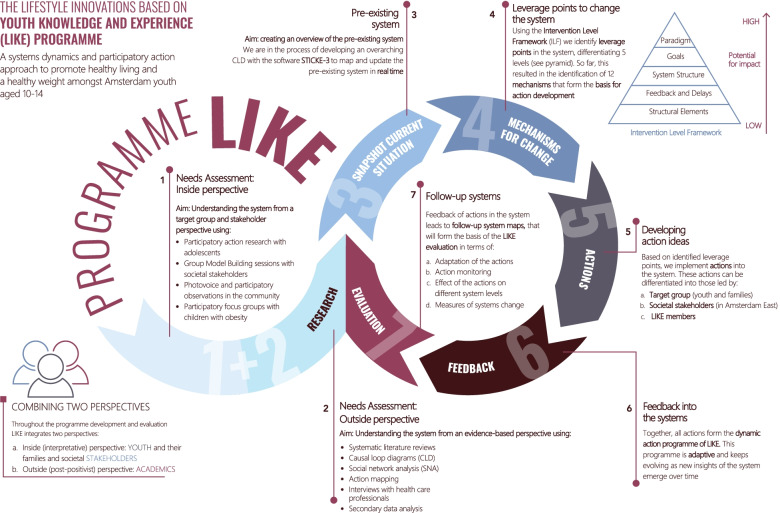
Overview of the LIKE programme

The LIKE programme is designed to tackle overweight and obesity in adolescents using a *system dynamics* and *participatory action research* approach [21]. It aims to develop, implement and evaluate a programme that will help transform the prevailing system into one that promotes healthy behaviour for adolescents (Table 3, Fig. 1).

**Table 3:** Key features of the LIKE programme

• The focus on the transition period from childhood to adolescence (age 10 to 14) is central to LIKE. • LIKE is a five-year programme (2018–2022) conducted in three lower socioeconomic neighbourhoods in the city of Amsterdam, Netherlands. • LIKE is part of the Amsterdam Healthy Weight Programme, a local-government-led whole-systems approach with the long-term goal of reducing childhood overweight and obesity in Amsterdam [22]. • In LIKE, we work in close collaboration with adolescents, families, societal stakeholders, researchers and local government to develop, implement and evaluate actions that will help transform the system into one where healthy behaviour is stimulated at the levels of child, family, neighbourhood, health care and city. For a more detailed description, we refer to the LIKE protocol paper [21].

In this step, the authors of the present study integrated the theoretical elements and evaluation stages of the six analysed systems evaluation studies and assessed how we could translate and apply those elements and stages to the development and evaluation of LIKE. Subsequently, using an iterative process, the original identified theoretical elements and evaluation stages (listed in Table 2) were checked to assess whether the emerging framework for the evaluation of LIKE was still in agreement with the included studies. Carrying out this step iteratively enabled us to combine the six systems evaluation studies with our experience in designing and evaluating LIKE. Worthy of note is that not all methods listed in Table 2 were included in the emerging framework for the evaluation of LIKE but only those methods considered appropriate to the context of LIKE. We thereby arrived at the ENCOMPASS framework, which can guide the evaluation of public health prevention programmes in complex adaptive systems in a comprehensive and practical way.

## Results

### The ENCOMPASS framework

**Fig. 2:**
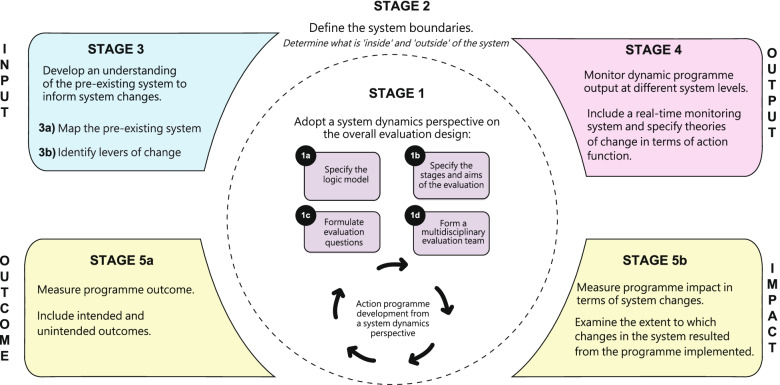
Overview of the various iterative stages in the ENCOMPASS framework^*^

The Evaluation of Programmes in Complex Adaptive Systems (ENCOMPASS) framework includes five iterative stages (Fig. 2) that reflect the components of a logic model as traditionally used in programme evaluation, including input (Stage 3), output (Stage 4) and outcome (Stage 5). These are preceded by stages of defining the evaluation research (Stage 1) and defining the system in question (Stage 2). In our description of each stage, we outline a theoretical underpinning with practical examples of how that underpinning is applied in LIKE and in other existing programmes in public health.

### Stage 1: Adopting a system dynamics perspective on the overall evaluation design

Most public health programmes, including those using a systems approach, follow a linear process of programme design, implementation and evaluation [23]. When one adopts a system dynamics perspective, however, such an approach does not work [24], because there are no pre-specified conditions, programmes or outcomes. Yet because such a linear process is standard practice, it is challenging not to fall back into the standard. In order to avoid that, the first stage in ENCOMPASS is to consciously adopt a system dynamics perspective and apply it throughout the evaluation – deliberately avoiding pre-specified outcomes and accepting the uncertainty that will bring into the evaluation. The *Lancet* Commission on Obesity, for instance, deviated from a mere focus on obesity as a specific outcome and instead reframed the problem as the ‘Global Syndemic’, including the much broader concepts of obesity, undernutrition and climate change [2]. Rather than focusing on specific determinants of those problems, such as knowledge about healthy food, the Commission applied a systems perspective to understanding the underlying drivers and the broad global outcomes that include human health and well-being, ecological health and well-being, social equity and economic prosperity. In LIKE, it proved challenging to find the right evaluation focus, and four sub stages were therefore formulated that could support that process: (a) specifying a logic model in system dynamics terms; (b) conducting each evaluation stage from a system dynamics perspective; (c) formulating research questions in system dynamics terms; and (d) forming the evaluation team.

#### Stage 1a: Specifying the logic model

In many public health evaluations, the logic model has a linear character from programme to impact, such as ‘We will assess whether a sugar tax (the programme) leads to higher prices for sugary drinks (output), leads to lower sugary drinks consumption (outcome), leads to lower BMI levels (effect/impact).’ In system dynamics terms, the evaluation is more dynamic, examining how the system is changing as the ‘event’ (programme) unfolds by looking at the emergent outcomes that result from interactions between the different elements of the system [25–27]. Although it is impossible to pre-specify a particular outcome when taking a systems approach, one will likely have a general theory about how the programme will lead to changes in the system. An example of that is given by Foster-Fisherman and colleagues [28], showing how a logic model in system dynamics terms does not focus on a distinct system part, such as broader pavements, with the expectation that this will result in the desired outcome, such as road safety. Instead, they highlight how systems contain a complex web of interdependent parts, where creating change in one part will lead to concurrent shifts in other parts of the system. Likewise, Sawyer and colleagues [22] formulated the logic model for the Amsterdam Healthy Weight Programme in system dynamics terms, hypothesising ‘that policy infrastructure and support for a whole-of-systems approach (input) permits integrated, cross-sectoral working (processes) to deliver targeted action (output) to elicit changes in the local environment (short-term outcomes), which increases children’s exposure to health-promoting environments (intermediate outcomes) and facilitates sustainable behaviour change through improved capability, opportunity, and motivation (individual level impact) and a transformed system underpinning childhood weight in Amsterdam (multilevel impact)’. The logic model in LIKE was similarly defined: ‘Understanding the system dynamics of obesity will help towards developing actions (theorising stage) that target the underlying mechanisms, thus leading to changes in the system (process evaluation stage) and ultimately to healthier environments, population health behaviours and BMI levels (impact evaluation stage)’ (see Fig. 1). It is evident that this logic model will require particular evaluation data (such as data on underlying mechanisms and system changes rather than on determinants and outcomes) [5]; this is explained in more detail in Stages 3 and 4.

#### Stage 1b: Specifying the stages and aims of the evaluation

In a systems approach, the logic model stages are iteratively intertwined; starting conditions, actions, and outputs, outcomes or effects also change over time. Stage 1b entails specifying the aims of the evaluation in system dynamics terms. In LIKE, the aims were defined as (a) to create an understanding of the system from multiple perspectives (rather than identifying the most important determinants of the problem); (b) to apply an adaptive programme development approach (rather than developing a fixed programme); to monitor action impact at different system levels (rather than measuring programme output); and to evaluate the overall impact of the programme on the system (rather than measuring programme impact).

#### Stage 1c: Framing evaluation questions

Once the evaluation aims have been specified, evaluation questions can be attached. That means formulating questions about starting conditions (Stage 3), about tracking system changes (Stage 4) and about programme outcome and the impact on system parameters (Stage 5). System evaluation questions should align with system dynamics concepts, and hence be phrased as ‘To what extent did the programme contribute to changes in the system?’ (rather than ‘Was the programme effective?’) [27, 29]. Furthermore, it is helpful to specify the types of epistemology, or theories of knowledge, that will be applied to understand, interpret and change the system [30, 31]. This is important because epistemologies will shape how researchers will interpret the evaluation data in terms of knowledge validity [32]. For example, a post-positivist epistemology, generally using quantitative data, may often be the preferred choice in evaluations, because it is believed to provide the most objective evidence on the effectiveness of programmes. On the other hand, an interpretive epistemology, which generally includes qualitative data, might help towards additionally understanding the specific context in which the study is taking place. This perspective acknowledges that there is no single reality, and that the complex nature of the system can be best understood by co-developing relevant knowledge in interaction between researchers and various groups of participants (see also Stage 3) [30].

Epistemological pluralism acknowledges that these multiple ways of knowing exist and may be equally valuable, and hence that embracing epistemological pluralism will result in a more thorough understanding of the complexity of the system. In other words, complex public health problems can then be understood from different angles [30, 32]. The questions specified by the *Lancet* Commission on Obesity [2] include issues framed in system dynamics terms such as Why do systems operate the way they do?; Why do systems need to change?; Why are systems so hard to change?; and What leverage points (or levers) are required to overcome policy inertia?. In LIKE, the research questions were formulated specifically for each of the different evaluation stages and were distinguished in terms of a post-positivist and/or interpretive epistemology, as shown in Table 4.

**Table 4:** Overview of LIKE evaluation questions

Evaluation stage	Related evaluation questions in the LIKE programme	Epistemology	
		**Post-positivist**	**Interpretive**
Theorising stage	1.1 What factors and processes in the pre-existing system in Amsterdam East shape unhealthy behaviours?	X	X
	1.2 From the perspective of adolescents/families/societal stakeholders, what factors and processes in the pre-existing system in Amsterdam East shape unhealthy behaviours?		X
	1.3 For those parts of the system that will be addressed by the action programme: What does the pre-existing system look like in terms of relevant stakeholders, power relations, and ongoing policies and activities?	X	X
Process evaluation stage	2.1 How has the understanding of the pre-existing system influenced the LIKE action programme?		X
	2.2 How is the action programme evolving, what action elements does it consist of, and what levels of the intervention level framework are being addressed by these actions?	X	
	2.3 What type of actions have resulted from the different approaches (group model building, participatory action research, mechanisms targeted by LIKE members) that we use?	X	
	2.4 How successful is the approach we follow in our LIKE programme in creating a sustainable programme, and how can this be optimised?	X	X
	2.5 How are actions monitored and adapted? And to what extent has this influenced the design and implementation of actions?	X	X
Outcome and impact evaluation stage	3.1 What type of (emergent, adapting, reinforcing) changes occurred in the living context, what were potential unintended consequences, and how can these be linked to the LIKE programme?	X	
	3.2 How do the target group and stakeholders perceive changes in the system and how do they perceive activities within the LIKE programme as contributing to these changes?		X
	3.3 To what extent was systems thinking incorporated in the LIKE action programme and in the work of relevant stakeholders?		X
	3.4 To what extent did the network of relevant stakeholders change because of the influence of LIKE?	X	
	3.5 What is the effect of the individual actions on the targeted action functions (that is, the specified theories of change)?	X	X

#### Stage 1d: Forming the evaluation team

A systems evaluation should be conducted by an evaluation team, ideally including different disciplines (which will aid towards embracing epistemological pluralism). The team is responsible for ensuring that a systems approach is applied throughout the programme design, implementation and evaluation. Active participation of team members throughout the entire programme is crucial in order for the evaluation team to develop an understanding of both the system and the programme. The team may, for instance, facilitate discussions and provide feedback amongst the programme members, in order to ensure that actions are being developed that can help disrupt the targeted mechanisms, or that actions are implemented at multiple system levels (Stage 4).

### Stage 2: Defining the system boundaries

Specifying the boundaries around the system is crucial, because they determine how a particular system is perceived, and hence define what is ‘inside’ and ‘outside’ the system [33, 34]. Appropriate boundaries can be based on two dimensions: (1) the programme’s purpose and (2) a determination of who and what is part of the system, given the targeted problem [28]. In setting the boundaries, it is important to realise that these are an artificial construct [7] and that selecting the appropriate boundaries is arbitrary and they may change due to external factors such as policy alterations or as the insights into the system progress [19]. Ways to guide the determination of system boundaries typically include the consideration that choosing overly tight boundaries could omit important feedback loops, because of the reductive effect of the boundaries [35]. Expanding boundaries may reveal important feedback loops in the system, but if the boundaries become too broad, the system cannot be adequately evaluated, because it will not be feasible to assess all its aspects [19, 35].

Foster-Fisherman and colleagues [28] provide examples for setting system boundaries in comprehensive community initiatives to tackle significant societal problems. Questions they asked include ‘Should the targeted system include local businesses and city institutions?’ and ‘Should it include residents from higher income neighbourhoods?’. They also noted the importance of specific attention to marginalisation, including non-traditional settings and individuals who are typically excluded from power and decision-making. Including such perspectives will provide different views of the system. In LIKE, the starting point for setting boundaries was the problem being targeted: encouraging healthy behaviour in the transition period from childhood to adolescence (age 10 to 14) in three focus neighbourhoods in Amsterdam. In terms of health behaviour boundaries, the focus was on four behaviours considered to be most relevant to the target group: physical activity, dietary behaviour, screen use and sleeping behaviour. In terms of system factors, we focused on social and physical environmental factors rather than on individual factors (such as psychological factors) or genetics.

### Stage 3: Understanding the pre-existing system to inform system changes

#### Stage 3a: Mapping the pre-existing system

The first part of the actual evaluation can now start: understanding the pre-existing system to inform system changes. Mapping the system is the ‘input’ in the logic model. For any evaluation approach, it is important to have some kind of basis for comparison (such as baseline data). In a systems evaluation, this translates to gaining an understanding of the pre-existing system, because there is no such thing as a baseline or a control system. The use of the prefix ‘pre-‘implies that there is a point in time that separates what is considered to be the previous system from the current system. Although the distinction in time can be marked by the implementation of a particular action, there is often no fixed point in time where programmes are implemented, because they adapt over time. Additionally, since the system itself adapts over time, an understanding of the pre-existing system cannot be captured at only one point in time. Therefore, understanding the pre-existing system is a process that can proceed up to and beyond implementation of the programme, because insights into how the system behaves are gained over time, including the responses to actions that are implemented in the system.

Understanding the pre-existing system includes: (1) insights into the separate system parts and the connections between them; (2) insights into the dynamics (or behaviour) of the system over time; and (3) insights into the system as a whole [12]. Understanding the pre-existing system usually involves a process of system mapping, such as the use of a causal loop diagram (CLD) or social network analysis (SNA) [5]. Feeding the content of these maps can be done on the basis of literature [36], expert opinions, participatory processes (as in group model building), or a needs assessment. Ideally, system mapping consists of a combination of multiple methods to gain an understanding of the pre-existing system from multiple perspectives. For example, in Change4Campbelltown, an Australian community-based systems approach to address childhood obesity, a CLD was developed by local leaders and community stakeholders in three locally facilitated community workshops. During the workshops, local drivers of childhood obesity were identified, along with the relationships between those drivers. The resulting diagram served as a logic model underpinning the design of co-designed activities that comprised the Change4Campbelltown initiative [37]. Another potential method includes SNA. McGlashan and colleagues [38] used this method to compare the role of stakeholder networks in two completed community-based intervention studies, Romp & Chomp (Australia) and Shape Up Somerville (USA). Results revealed how this method can be used to quantify the structure and strengths of the interpersonal networks between stakeholder members during the intervention period.

In LIKE, the aim was to develop an understanding of the pre-existing system from a post-positivist and an interpretive perspective [21]. To achieve this, a mixed-methods needs assessment was conducted, with data collected from adolescents, families and societal stakeholders using participatory action research, focus groups, participant observations and group model building (Fig. 1). These data were complemented with data from a post-positivist perspective using systematic literature reviews and CLDs (published elsewhere) [36]. Moreover, SNA was performed to map relevant actors in the system and their relationships, and to track changes over time (see also Stage 5). These needs assessment data were combined into an overarching CLD showing the pre-existing system as a whole (results will be published elsewhere).

#### Stage 3b: Identifying levers of change

To aid in moving from a systems map to understanding the system dynamics, there are a number of frameworks available [7, 39, 40]. These originate from the work of Meadows and Wright, who, in their framework on finding leverage points in the system, identified twelve places in the system where one can intervene – *leverage points* – to produce system changes, ranging from system parameters such as numbers up to the system’s paradigm [7]. These twelve places were summarised into five, more mutually exclusive, levels, through the use of the Intervention Level Framework (ILF) proposed by Johnston and colleagues [39]. This framework was developed with the aim of providing researchers support in finding solutions to complex public health problems. The highest level in this ILF is the system’s deepest-held belief, or *paradigm;* intervening at this level will produce the most disruptive changes in the system. An example is ‘Healthy food should be available, accessible and affordable for everyone in current and future generations’ [41]. The second level is *goals,* and actions targeting that level can modify the aim of a system, for example ‘food prices that reflect the costs of toxic exposure, environmental clean-up, and depletion of natural resources’ [41]. The third level is *system structure,* which describes the interconnections between the various elements of the system and subsystems, for example ‘Incorporate more fresh food into school meals by connecting local growers to schools’ [39]. The fourth level is *feedback and delays,* which enables self-regulation of a system by relaying information about outcomes of actions back to the source of actions, for example ‘Evaluate sales taxes on less healthy, energy-dense foods’ [39]. The fifth level, and the easiest level in which to intervene, involves the *structural elements* such as actors, subsystems and physical elements of a system [39]. An example would be implementing front-of-pack nutrition labelling to encourage healthier food purchases [41]. Applying the ILF ensures developing an understanding of the different system levels, as well as gaining an understanding of the deeper system structures. More recently, the Action Scales Model was developed with the aim of providing practitioners and policymakers (rather than researchers) with a practical tool to help them identify entry points for action [40]. In the paper in question, Nobles and colleagues showed how application of the different system levels has three primary uses for practitioners and policymakers: (a) to help understand how the system works, and explain why the system generates the outcomes it does; (b) to facilitate finding leverage points for system changes, and (c) to ensure that there is coherence among actions being implemented [40].

Following the initial understanding of the pre-existing system, including identifying potential levers of change, a programme can be developed and implemented in an iterative process of reflection and adaptation, composed of actions that target the various levers with the aim of transforming the system into the desired state. The process of action development in complex systems will be explained in a separate paper, but important concepts for evaluation of those actions are detailed below in Stage 4.

Brown and colleagues [42] used system mapping and analysis to propose a theory of change for community-based programmes aiming to build capacity for obesity prevention. They showed that key elements of systems approaches include community involvement, collaboration, quality of action, feedback about programme success, research support, and how leadership interacts with community health behaviours and outcomes. McGlashan and colleagues [43] applied network analytic methods, similar to SNA, as a new way to gain quantitative insight into the structure of an obesity CLD to inform programme design. In LIKE, leverage points are identified from a bottom-up (interpretive) and top-down (post-positivist) perspective. Bottom-up actions are developed by the local-stakeholder action groups. Top-down action development is guided by the pre-existing system map and by application of the Intervention Level Framework to identify leverage points at each of the five system levels (see Table 5).

**Table 5:** Steps for the identification of leverage points in the LIKE programme

What is the target problem?	Step 1. What is the direct cause of this problem (that is, the tip of the iceberg)?	Step 2. What are the underlying mechanisms causing this problem?	Step 3. What are potential leverage points (at the ILF levels)?
In the transition period from primary to secondary school, adolescents make less use of outdoor facilities (such as parks and sport fields). They are therefore less physically active in their spare time.	Most outdoor facilities are designed for young children. Adolescents therefore do not find such environments attractive. The absence of peers at the facilities further inhibits their use of them.	Adolescents do not participate in decision making regarding the design of outdoor facilities. The facilities are therefore unattractive for them to use.	1. Paradigm: Active outdoor play is considered a routine behaviour among adolescents. 2. System goals: The system serves the needs of adolescents. 3. System structure: Urban design planners and youth representatives collaborate on a regular basis. 4. Feedback and delays: Outdoor facilities are attractive, and adolescents therefore make more use of them. Because more adolescents use these facilities, that becomes more attractive for other adolescents. 5. Structural elements: Secondary schools encourage adolescents to use outdoor facilities.

### Stage 4: Monitoring dynamic programme output at different system levels

Evaluation of public health programmes generally includes a process evaluation (whose purpose is to explain how a programme has generated outcomes and effects). Such an evaluation is necessary for a thorough understanding of the implementation process, as well as of the contextual factors and mechanisms that underlie observed changes following a programme. This will enable a wider application of a particular programme in real life [25]. A process evaluation from a complex system dynamics perspective goes beyond the consideration of contextual or implementation factors. It also seeks to monitor how the wider system responds to a programme, thus enabling programme adaptation on the basis of the information obtained [5]. To this end, this type of evaluation has a number of specific characteristics in terms of individual action specification and the monitoring of the programme as a whole. First, evaluating programme output from a system dynamics perspective requires a monitoring system with real-time feedback, so that action and system feedback can be tracked across time [37]. A real-time monitoring system helps in capturing adaptations of individual actions, as well as in tracking how the programme as a whole evolves over time.

At the action level, it is important to specify a theory of change in terms of the function of the action, and then to decide on measurements that align with that theory of change. If, for example, an action function is to involve children as the group targeted for empowerment in the action development process, then the process evaluation should measure levels of child empowerment. In formulating outcomes, it is important to keep in mind both intended and unintended consequences, because the latter are also of help in building the system maps.

At the programme level, it is important to capture data that can be used to monitor the programme as a whole. Most importantly, data should aid in monitoring whether actions are targeting the ‘right’ levels of the system (because the higher the level targeted, the greater the likelihood of transforming the system). Individual actions should therefore specify what ILF system level they are targeting. Second, depending on the programme’s aims, the different system parts that the actions are targeting – such as the various settings or behaviours – should be tracked. Together, the information at action and programme level can be used to adapt or complement the various actions – for instance the need to include actions that target ‘higher’ system levels [37]. For example, the process evaluation in the earlier mentioned Change4Campbelltown programme included an action register, a stakeholder engagement database, and key engagement activities, with data collected quarterly over 2 years of reporting [37]. Actions documented in the implementation register were represented on the CLD made at the start of the programme (that is, the pre-existing map). Properties for each action were collected, including its status (active, not active) at each quarter, and to which of the themes from the CLD it was primarily connected.

In LIKE, in order to monitor action output, plenary meetings with all action groups are held every 6 weeks to discuss action progress. Groups are requested to keep track of information regarding action development (facilitators, barriers, which actions have made it to the implementation stage, or why not) as well as characteristics of the actions (ILF level, setting, target behaviour, stakeholders involved). To track progress, actions are added to the pre-existing system map developed in Stage 3. That way it is possible to visualise where actions are situated in the overarching CLD; which ILF levels, settings and behaviours they are targeting; and hence where action is lacking (details on this process will be published elsewhere).

### Stage 5: Measuring programme outcome and impact in terms of system changes

#### Stage 5a: Programme outcome

As the programme progresses, evaluation data will increasingly contain, besides information on output, information about the outcomes of actions and about intended and unintended consequences, based on the specified theories of change. Such information supports the programme outcome stage. For example, if the function of an action is to connect local food businesses to empower them in facilitating healthier food environments, then information on the healthiness of those food environments will ultimately be needed for the evaluation. Such information on programme outcomes can then be included in the system maps (CLDs). At systems level, CLDs can be compared over time to see how various parts in the system are changing in terms of outcomes as a result of the actions being implemented (see Stage 5b). To track changes in the system, indicators can be developed based on the theories of change specified for the actions, whereby indicators serve as a proxy for outcome measurements; these allow for measurements at different points in time that enable the evaluation to capture changes in the system occurring in various stages. Such indicators should be dynamic and should be developed once actions have been implemented; only then will a better understanding of the system be developed. Hennessy and colleagues [20], for example, created a retrospective systems map representing the community change dynamic within the Shape Up Somerville programme. The systems map had two components: (1) qualitative and quantitative data collected during the programme stage and (2) qualitative data generated by the research systems mapping team during group discussions and interviews. Questions included ‘During the 10-year period of Shape Up Somerville, what did you observe: Trends? What set of interrelationships caused or are causing those trends? What were the dampening effects? How long did the various changes take to gain momentum?’. The systems map was revised after each group discussion or interview.

#### Stage 5b: Programme impact

In Stage 1c, we argued that the main evaluation question in a systems evaluation should be phrased as ‘To what extent did the programme contribute to changes in the system?’. In Stage 5, the aim is therefore to clarify *contribution* (‘How reasonable is it to believe that the programme effectively contributed to the intended goals?’*)* and not to identify *attribution* (‘What proportion of the outcomes was produced by the programme?’) [21, 44]. Here, ‘contribution’ involves looking not only at how the actions change the targeted feedback loops, but also at how the perturbations caused by those actions positively or negatively affect other feedback loops or subsystems [45]. Information on programme output and outcome, including unexpected outcomes, has been collected as part of Stages 4 and 5a. This final stage, Stage 5b, therefore views all changes as a whole and examines the extent to which changes in the system have resulted from the programme implemented, rather than from external factors.

Looking at changes in the system as a whole involves comparing the system maps developed in the earlier stages over time (such as CLDs or SNA). To measure contribution, the changing system maps can be presented to the various stakeholders to obtain their interpretations of the system changes. This is because the determination of whether a change has, or has not, taken place due to a programme’s contribution ultimately depends on whose perspective is taken into account. Sensemaker, for example, is a complexity-aware, narrative-based methodology that recognises that people make sense of their world based on their experiences [46]. In LIKE, ‘contribution stories’ from our target groups (adolescents, families and societal stakeholders) are used to assess how they perceive the contribution of LIKE to system changes. The system maps created during the programme are used as a starting point in those contribution stories, and participants have opportunities to update or adapt those maps, depending on how they perceive programme impact [21]. Because we embrace epistemological pluralism in a systems evaluation, multiple methods should ideally be applied in order to determine programme contribution.

Finally, when assessing contribution, it is also important to take into account the timing of the evaluation, since system changes occur at different paces. The programme being evaluated may have made a significant contribution to achieving a particular system change (like a national policy that bans online marketing of unhealthy food); however, the programme evaluation may have ended just before the ‘tipping point’ (critical point in which a system shifts towards a different state) was reached; the evaluation may thereby underestimate the longer-term impact of the programme [47]. Ultimately, the evaluation timing becomes a search for the right balance between what the evaluation goal is and what is possible: conducting the evaluation until system changes are achieved versus the availability of financial support and capacity for continuing the evaluation. One way to address this issue of time is to specifically distinguish between expected short-, medium- and long-term outcomes and how each part of the evaluation will contribute to each outcome.

An example of capturing the wider impact of system changes over time can be seen in the use of ripple effects mapping, as explained by Nobles and colleagues [48]. This method can be used to identify the wider intended and unintended impacts of a programme in a system and to understand some of the mechanisms – and chains of events – that might explain why a programme produces the impact(s) that it does. In LIKE, we will use ripple effects mapping to capture both the intended and unintended consequences of the actions that were undertaken by the various stakeholders, as well as consequences of the programme as a whole. Recent experience from the Amsterdam Healthy Weight Approach showed that these types of learnings could include, as core working principles, the articulation of responsive adaptation, a learning approach, multi-level action and cross-sectoral working, and representations of the relationship between the programme and the system in which it operates [22].

## Discussion

This paper has outlined the ENCOMPASS framework (Fig. 2), which can guide the evaluation of public health programmes in complex adaptive systems. The framework consists of five iterative stages: (1) adopting a system dynamics perspective on the overall evaluation design; (2) defining the system boundaries; (3) understanding the pre-existing system to inform system changes; (4) monitoring dynamic programme output at different system levels; and (5) measuring programme outcome and impact in terms of system changes (Fig. 2). The value of ENCOMPASS lies in the integration of key characteristics from existing systems evaluation studies, as well as in its practical, applied focus. It can be applied to the evaluation of public health programmes in complex adaptive systems, all the way from understanding the system, to developing actions to change the system, to measuring system changes.

Although systems thinking in public health has been advocated for more than a decade, the application of system methodologies in public health is still in its infancy [5]. This applies not only to the development of programmes, but certainly also to their evaluation. Should a general consensus be reached on the methodological requirements to be set for the development, implementation and evaluations of programmes in complex adaptive systems, this consensus could contribute to a certain degree of quality control and thereby help accelerate knowledge production in understanding complex adaptive systems. This is exactly what was aimed for when creating ENCOMPASS. Although it is too early for full consensus to be reached, EMCOMPASS provides a first step in facilitating researchers in evaluating public health programmes from a systems perspective. ENCOMPASS can be seen as a living document that can be adapted as insights develop over time. As researchers apply ENCOMPASS, a number of issues are important to consider.

First, the five stages of ENCOMPASS have been described as if they form a linear process. In practice, however – just as is broadly advocated in evaluation theory – evaluations of programmes in complex adaptive systems will also always involve an iterative process. For example, boundaries may need to be adjusted as a programme develops over time, which in turn might necessitate adjustment of the evaluation questions. Moreover, insights that are being gathered during the evaluation process will likely change insights into the (pre-existing) system which in turn might lead to the development of new actions. Researchers that intend to use ENCOMPASS should therefore be aware that the various stages described in this framework may be carried out multiple times and in different sequences. The dynamic nature of this type of evaluation is also the reason why we have designed the overall structure of LIKE as a continuous process, whereby the outcome of previous stages feeds into the stages that follow (Fig. 1). Partly due to this iterative process, it is not easy, in our experience, to assess how much time each stage will take and where exactly each stage ends and a new stage begins.

Second, in the development of ENCOMPASS it has become clear that the *evaluation* of public health programmes from a systems perspective cannot be easily separated from the process of *developing* programmes; largely because the programme being developed is not static and keeps adapting based on the results of the preliminary and ongoing evaluation (i.e., as insights into the pre-existing system emerge). While the development of programmes in complex adaptive systems is beyond the scope of the current study, it is important to mention this here because it implies that one cannot carry out the evaluation in isolation or wait until the end when the programme is completed. Similarly, the programme stakeholders involved are not merely the recipients of the evaluation outcomes at the end. Rather, the researcher forms a team with the stakeholders involved in the development, implementation and evaluation of the programme, as well as with other actors in the system that the programme aims to change [26]. The provision of real-time feedback between research, policy and practice ensures that the various actors can reflect on and adapt their programme over time and form an important element of the ENCOMPASS framework. The actual resources that are necessary to successfully carry out the evaluation (such as time, money, leadership and willingness to adapt) therefore require specific attention.

The primary aim of the ENCOMPASS framework is to provide a first concrete guide in supporting researchers in designing evaluation research on programmes in complex adaptive systems. By integrating existing systems evaluation studies within the ENCOMPASS framework, and by offering examples of how programmes in complex adaptive systems can be evaluated in practice, ENCOMPASS contributes to a more uniform synergy between systems thinking and evaluation practice in the public health sector. Although the authors of the current study acknowledge that fully adhering to each stage as described in this framework is ambitious, we believe that the general principles form an important stepping stone towards integrating systems thinking into public health research and practice, even when programmes manage to include only parts of the stages. Ultimately, this can further the ambition of the systems perspective to generate public health evidence that better accounts for the complexity of the real world.

### Limitations of ENCOMPASS

We can identify two important limitations of the ENCOMPASS framework. First, we have limited the studies on which ENCOMPASS is based to studies in public health. The integration of systems thinking and evaluation is also discussed, however, in other fields such as economics and ecology. These fields may also provide practical frameworks that can be used for systems evaluation. Integration of such a multi-disciplinary perspective would be important in future studies. Second, ENCOMPASS was developed by integrating existing public health systems evaluation studies and by iteratively applying this systems evaluation theory to the evaluation of the LIKE programme. However, LIKE is still in its early evaluation stage, which means that not all the stages described in ENCOMPASS have so far been put into practice. At this moment, it is also not known what the full evaluation process would yield in terms of results. Moreover, ENCOMPASS has not yet been tested in the evaluation of any other programme than LIKE. As several iterations have already been made to the ENCOMPASS framework during the evaluation process so far, it is expected that the framework will further develop as it has been applied more extensively. Ideally, in future studies, it would be valuable to evaluate ENCOMPASS itself as a framework for evaluating programmes in complex adaptive systems and assess: (a) whether the theory described in each stage is indeed applicable in practice; (b) what types of results each stage yields; and (c) how various stakeholders perceive the value and validity of the results.

## Conclusions

This paper integrated key characteristics of existing system evaluation studies in public health and applied these characteristics to the context of the LIKE programme. This process resulted in the development of the ENCOMPASS framework, which contains five iterative stages: (1) adopting a system dynamics perspective on the overall evaluation design; (2) defining the system boundaries; (3) understanding the pre-existing system to inform systems change; (4) monitoring dynamic programme output at different system levels; and (5) measuring programme outcome and impact in terms of system changes. Although, by the nature of systems thinking, the ENCOMPASS framework is likely to further mature over time, this framework brings value in providing researchers first practical guidance into initiating systems thinking in public health – as well as in how to evaluate programmes in complex adaptive systems in a practical way. ENCOMPASS thereby contributes towards developing better evaluation standards and practices that can in turn generate evidence that accounts for the complexity of the real world and improve health.

## Data Availability

The datasets used and/or analysed during the current study are available from the corresponding author on reasonable request.

## Abbreviations

CLDs: Causal loop diagrams
ENCOMPASS: Evaluation of Programmes in Complex Adaptive Systems
ILF: Intervention level framework
LIKE: Lifestyle Innovations Based on Youth Knowledge and Experience programme
SNA: Social network analysis
